# Toscana Virus Infection in American Traveler Returning from Sicily, 2009

**DOI:** 10.3201/eid1609.100505

**Published:** 2010-09

**Authors:** Meagan K. Kay, Katherine B. Gibney, Francis X. Riedo, Olga L. Kosoy, Robert S. Lanciotti, Amy J. Lambert

**Affiliations:** Author affiliations: Centers for Disease Control and Prevention, Atlanta, Georgia, USA (M.K. Kay);; Centers for Disease Control and Prevention, Fort Collins, Colorado, USA (K.B. Gibney, O.L. Kosoy, R.S. Lanciotti, A.J. Lambert);; Evergreen Hospital Medical Center, Kirkland, Washington, USA (F.X. Riedo)

**Keywords:** meningitis, sandfly, Toscana virus, vector-borne infections, Sicily, travel medicine, letter

**To the Editor:** Since the discovery of Toscana virus (TOSV) in 1971 in Tuscany (*1*), sandfly-borne TOSV has become recognized as a leading cause of acute meningitis in central Italy during the summer (*2*). France, Spain, Portugal, Greece, and Cyprus have also reported cases of TOSV infection (*2*). Although TOSV has been detected in sandflies in Sicily (*3*), we are not aware of any historically documented human infection with TOSV in this southernmost region of Italy.

We report TOSV infection of an American male physician, 65 years of age, who traveled to Sicily for 3 weeks and returned to the United States in October 2009. Two days after his return, he awoke with a headache, and hours later he noticed difficulty finding words. His headache progressed, and during the next few hours, he experienced severe expressive dysphasia. At admission to the hospital, he denied having fever, nuchal rigidity, photophobia, nausea, vomiting, or diarrhea.

Other than changing planes in Milan, the patient had remained in Sicily during the entire 3 weeks of his visit. He had sustained both mosquito and what he thought were flea bites while in Sicily. He had no known exposure to bats, rabid animals, or ticks.

Computed tomographic scan and magnetic resonance imaging of the brain showed no mass lesions or abnormality of the cerebral vessels. A sample of cerebrospinal fluid (CSF) obtained at admission showed 14 leukocytes/mm^3^ (reference range 0–5 leukocyte/mm^3^) with 100% lymphocytes, a protein level of 126 mg/dL (reference range 15–45 mg/dL), and a glucose level of 63 mg/dL (reference range 50–80 mg/dL). A nasopharyngeal swab specimen was negative for influenza A and B virus antigens. Other than a decreased thrombocyte count and an elevated serum glucose level, the results of complete blood count, comprehensive chemical panel, and coagulation studies were within normal limits. PCR results for CSF were negative for herpes simplex virus, enterovirus, and parechovirus. Test results for acute-phase and convalescent-phase serum specimens performed at the Washington State Department of Health Laboratory were negative for West Nile virus and St. Louis encephalitis virus immunoglobulin M.

Serum and CSF were sent to the Centers for Disease Control and Prevention in Fort Collins, Colorado. TOSV RNA was detected in a CSF sample collected on day 1 of illness by using reverse transcription–PCR (*4*). Plaque-reduction neutralization assays demonstrated a >4-fold rise in TOSV neutralizing antibodies between paired serum specimens collected on days 1 (titer <1:10) and 21 (titer 1:320) of illness. No similar rise in neutralizing antibodies to serologically related phleboviruses (e.g., sandfly fever Naples virus and sandfly fever Sicilian virus) was detected. The patient received supportive care only. He had a complete neurologic recovery in 10 days and was able to return to work.

Phylogenetic analyses indicate that 2 geographically distinct genotypes, the Italian and Spanish lineages of TOSV, circulate throughout the Mediterranean region (*5*). To determine the lineage of the infecting strain, we performed advanced molecular analyses of TOSV RNA isolated from the infected traveler’s CSF. These analyses used published consensus primers that target the small (S) segment (*4*) as well as primers newly designed to target the medium (M) segment: M 851F, 5′-ACCAAATACAACCATAGCCCC-3′ (forward) and M 1327c, 5′-ATACAATTCCCACAGTCGTTAG-3′ (reverse) of the multisegment TOSV genome. Reverse transcription–PCR amplification and nucleotide sequencing generated 2 nt sequences of 332 (S segment) and 424 (M segment) nucleotides in length. Phylogenetic analyses of the newly determined sequences and sequences previously determined for Mediterranean TOSV isolates of diverse origin were carried out by using MEGA version 4 (*6*). According to phylogenetic inference, the TOSV RNA identified in the returning traveler is of the Italian lineage (Figure). Of interest, the TOSV M segment sequence generated from this patient aggregates with extreme bootstrap support along with that generated previously from a strain of TOSV that was isolated from sand flies in Palermo, Sicily, in 1993 (Figure), indicating that the infecting strain is likely representative of strains that have circulated in Sicily for years.

**Figure:**
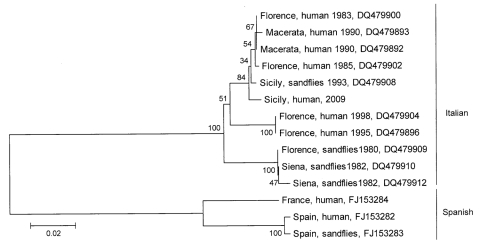
Phylogeny of Toscana viruses (TOSVs) of diverse origin. Partial small (S) and medium (M) segment sequences of interest were aligned by using ClustalW (www.ebi.ac.uk/Tools/clustalw2/index.html), and neighbor-joining and maximum-parsimony trees were generated by using 2,000 bootstrap replicates with MEGA version 4 (*6*). Highly similar topologies and confidence values were derived by all methods, and a neighbor-joining tree generated from a comparison of 424 nt of the M segment polyprotein gene open reading frame is displayed here. GenBank accession numbers appear after the location and source of isolation for each taxon. Scale bar represents the number of nucleotide substitutions per site. Of interest, the 2009 Sicilian TOSV described in this study (Sicily, human, 2009) aggregates with extreme support along with other Italian viruses, including an isolate that was derived from sandflies in Palermo, Sicily, in 1993.

This case represents the third report of meningitis or meningoencephalitis caused by TOSV infection in a US traveler to the Mediterranean (all acquired in Italy) (*7**,**8*). As is shown by this and other recent reports of TOSV infections in the Mediterranean islands surrounding Italy (*9*), the geographic range of TOSV human infections is larger than previously known. Reports of TOSV infection among European travelers returning from disease-endemic regions have provided additional evidence of the emergence of TOSV-related illness on a global scale (*10*).

Although the clinical course varies from asymptomatic infection to severe meningoencephalitis, TOSV should be included in the differential list of viral pathogens among patients who seek treatment with symptoms consistent with meningitis or encephalitis if the patients have recently traveled to Mediterranean areas, including Sicily. Because neither a vaccine nor specific antiviral drug treatment is available to prevent or treat TOSV infection, travelers to TOSV-endemic areas should be advised to take all precautions to prevent insect bites.
